# Host-Pathogen Interactions Made Transparent with the Zebrafish Model

**DOI:** 10.2174/138945011795677809

**Published:** 2011-06

**Authors:** Annemarie H Meijer, Herman P Spaink

**Affiliations:** Institute of Biology, Leiden University, Einsteinweg 55, 2333 CC, Leiden, The Netherlands

**Keywords:** Bacterial infection, chemokine receptors, *Danio rerio*, embryo model, high-throughput drug screening, innate immunity, Toll-like receptors, tuberculosis.

## Abstract

The zebrafish holds much promise as a high-throughput drug screening model for immune-related diseases, including inflammatory and infectious diseases and cancer. This is due to the excellent possibilities for *in vivo* imaging in combination with advanced tools for genomic and large scale mutant analysis. The context of the embryo’s developing immune system makes it possible to study the contribution of different immune cell types to disease progression. Furthermore, due to the temporal separation of innate immunity from adaptive responses, zebrafish embryos and larvae are particularly useful for dissecting the innate host factors involved in pathology. Recent studies have underscored the remarkable similarity of the zebrafish and human immune systems, which is important for biomedical applications. This review is focused on the use of zebrafish as a model for infectious diseases, with emphasis on bacterial pathogens. Following a brief overview of the zebrafish immune system and the tools and methods used to study host-pathogen interactions in zebrafish, we discuss the current knowledge on receptors and downstream signaling components that are involved in the zebrafish embryo’s innate immune response. We summarize recent insights gained from the use of bacterial infection models, particularly the *Mycobacterium marinum* model, that illustrate the potential of the zebrafish model for high-throughput antimicrobial drug screening.

## INTRODUCTION

The discovery of effective antimicrobial drugs has contributed to major gains in life expectancy, but, infectious diseases are still the major cause of death in developing countries and a world-wide threat is posed by increasing antibiotic resistances of pathogens. For example, human tuberculosis is responsible for close to two million deaths per year and one-third of the world population carries a latent tuberculosis infection [1]. New drugs to combat this disease are urgently needed due to the evolution of multi drug resistant (MDR) and extensively drug resistant (XDR) strains. The causative agent of human tuberculosis, *Mycobacterium tuberculosis*, is able to outwit many aspects of the immune system and can persist for many years in specialized structures of infected and non-infected immune cells, called granulomas [1]. The complex interactions that *M. tuberculosis* and many other pathogens have with their hosts, explains why drugs that may target the bacteria *in vitro* are often not effective *in vivo*. Therefore, novel drug development strategies to combat infectious diseases will be most effective when conducted using animal models.

**Fig. (1):**
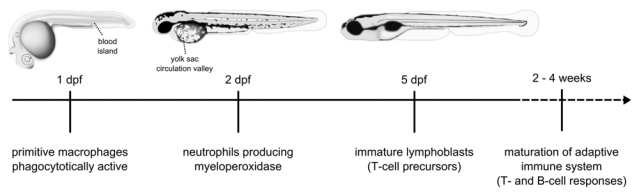
Schematic overview of the development of the zebrafish immune system. Commonly used sites for systemic bacterial infection of embryos by microinjection are the blood island and the yolk sac circulation valley at 1-3 dpf.

The zebrafish holds much promise as a high-throughput drug screening model [2]. In the recent years, zebrafish models for studying human pathogens or closely related animal pathogens have emerged at a rapid pace, either using adult fish with a fully developed adaptive immune system, or using embryos or larvae that rely solely on innate immunity (Table **1**) [3-8]. The zebrafish and human immune systems are remarkably similar, as discussed in several other reviews [9-12]. The main strength of the zebrafish as a vertebrate model for studying infectious diseases lies in the excellent possibilities for *in vivo* imaging of host-pathogen interactions in combination with advanced tools for genomic and large scale mutant analysis. For this reason, many infectious disease studies in the zebrafish model have concentrated on the embryonal and larval periods of development, when the advantages of the model are maximal. A single pair of fish can produce hundreds of offspring every week. The embryos develop externally and remain transparent during several days of larval development. Establishing methods to rear embryos under germ-free or gnotobiotic conditions has been an important contribution for studying host-microbe interactions in a controlled environment [13, 14]. Already after one day of embryogenesis, the embryos possess functional macrophages that are capable of sensing and responding to microbial infections [15]. The context of the embryo’s developing immune system makes it possible to study the contribution of different immune cell types to host-pathogen interaction (Fig. **1**). For example, infections can be performed before (at 1 day post fertilization (dpf)) or after the presence of differentiated neutrophils (at 2 dpf) [16]. The behavior of the embryo’s immune cells can be tracked using video-enhanced differential interference contrast (DIC) microscopy [17] or using transgenic lines with fluorescently marked immune cell populations as discussed below [18]. With the zebrafish embryo model, the dynamics of fluorescently labeled proteins in a living vertebrate organism can now be studied even at single-molecule level [19].

**Fig. (2):**
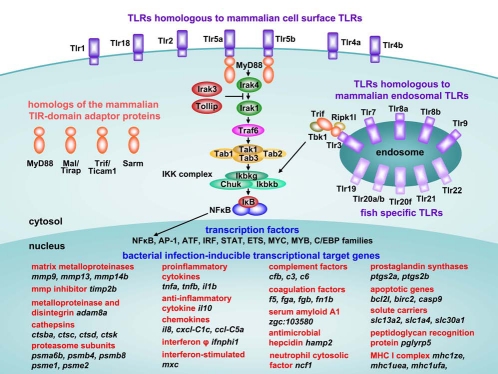
Components of the TLR pathway and genes commonly induced during the innate immune response of zebrafish to bacterial infection. Annotation of the zebrafish TLRs is based on Meijer *et al*. [118]. Cell surface or endosomal localizations of the zebrafish TLRs are hypothetical, based on localization of their mammalian homologs. The fish specific TLRs are tentatively placed on the endosome, since members of this group have been shown to recognize DNA or RNA ligands similar to the mammalian endosomal TLRs [169, 170]. The zebrafish genome encodes four TIR-domain adaptor proteins and lacks a homolog of the mammalian TRAM/Ticam2 adaptor [118-120]. Zebrafish Tlr5a/b and mammalian TLR5 share specificity for the recognition of bacterial flagellin [26]. The pathway from Tlr5a/b to NFκB is based on data from mammalian models [116] and has not been experimentally confirmed in zebrafish. The pathway from Tlr3 to NFκB is based on Sullivan *et al*. [119]. A selection of common transcriptionally-induced target genes is shown, based on *S. typhimurium* infection experiments in zebrafish embryos [26, 30] (unpublished results). Many of these genes are also induced in adult zebrafish at the end stage of *M. marinum* infection [25, 27, 28]. The indicated transcription factor families are induced by TLR signaling or by other pathways involved in the innate immune response. Several members of these transcription factor families as well as several components of the TLR pathway also show induced expression levels during bacterial infections [25-28].

The small size of zebrafish adults (3-5 cm), the high population density (5 fish/liter) at which they can be kept, together with their high reproductive capability have made the zebrafish the vertebrate model of choice for forward genetic screens. These screens have resulted in the identification of many genes relevant to human development and disease [20] and the first successful application of forward genetics in studying infectious disease was recently reported [21]. The availability of the zebrafish genome sequence and efficient tools for reverse genetics also contribute to the usefulness of the model [22-24]. Microarray and deep sequencing data sets have provided insights into the zebrafish transcriptome during infections and are powerful tools to provide leads for functional studies (Fig. **2**) [25-30]. Finally, the millimeter size of the zebrafish embryos and larvae makes them highly suited for screening chemical libraries, with the advantage that compounds can be administered simply to the embryo medium and that only minute quantities are needed [2]. 

A growing list of bacterial and viral pathogens has been used for experimental infections in zebrafish, as detailed in several excellent reviews [3-5, 7, 11]. Zebrafish are also susceptible to parasitic infections [31, 32], and recently, also fungal infection models have been established, where optimal advantage of the zebrafish’ poikilothermic physiology can be taken for studying temperature-dependent fungal dimorphism [8, 33]. In this review we will focus on bacterial infection models (Table **1**). Following a brief overview of the zebrafish immune system and the tools and methods used to study host-pathogen interactions in zebrafish, we will discuss the current knowledge on receptors and downstream signaling components that are involved in the zebrafish embryo’s innate immune response, and will discuss recent insights gained from the use of bacterial infection models, particularly the *Mycobacterium marinum* model. We will also discuss the potential of the zebrafish model for high-throughput antimicrobial drug screening strategies.

## CHARACTERISTICS OF THE ZEBRAFISH IMMUNE SYSTEM

Adult zebrafish have innate and adaptive branches of the immune system similar as in mammals or all other jawed vertebrates [9, 10]. However, innate immune functions can be studied in separation from adaptive functions in zebrafish embryos, since lymphoid cells only develop later during larval stages (from 4 dpf) and since the adaptive immune system is not fully matured until approximately 4 weeks post fertilization [34] (Fig. **1**). Most if not all cell types of the mammalian immune system have been identified in zebrafish or other teleost fish species [11, 32, 35], including most recently a subset of antigen-presenting cells strongly resembling the mammalian dendritic cells [36]. Like in mammals, the development of immune cells during embryo-genesis occurs in distinct waves of primitive and definitive hematopoiesis [37-40]. Hematopoiesis shifts several times between different locations in developing embryos and larvae [39, 40]. Despite differences in the sites of hematopoiesis between zebrafish and mammals, the cellular and regulatory processes of hematopoiesis are highly conserved [35, 39, 40].

The primitive wave of hematopoiesis initiates at two distinct sites during the first day of embryogenesis. At the anterior lateral plate mesoderm or rostral blood island hemangioblasts differentiate into myeloid cells, while the posterior lateral plate mesoderm, which later forms the intermediate cell mass, give rises to erythroid cells [37, 39, 40]. A transient wave of hematopoiesis occurs in the posterior blood island of 1 day old embryos that contains erythromyeloid progenitor cells (EMPs), the first multipotent hematopoietic progenitor cells [38, 41]. The posterior blood island region later expands into the caudal hematopoietic tissue, which forms a transient site of hematopoiesis analogous to the mammalian fetal liver [42]. As in mammals, a wave of definitive hematopoiesis starts in the aorta-gonad-mesonephros (AGM). Important breakthroughs in zebrafish stem cell research for the first time showed the transition of aortic endothelial cells into blood cells using time-lapse confocal imaging of zebrafish embryos [43, 44]. Comparison with studies in *ex vivo* slices of mouse embryos demonstrates a remarkable conservation of this process in all vertebrates [45]. By lineage tracing studies it has been shown that blood cell precursors arising from the AGM seed the caudal hematopoietic tissue, from where cells migrate to the thymus and pronephros [42, 46, 47]. In addition, direct migratory routes from the AGM to the thymus and pronephros have been demonstrated [47, 48]. The pronephros develops into the kidney marrow in adult fish and is considered as the equivalent of the mammalian bone marrow [9]. Although zebrafish have no lymph nodes, there is evidence of the development of a lymphatic system between 3 and 5 dpf [49, 50].

The transcriptional regulation of hematopoiesis appears largely conserved among vertebrates, and a number of zebrafish orthologues of crucial mammalian transcription factors have been studied, for example members of the RUNX, C/EBP and ETS families [40]. The ETS transcription factor Spi1, also known as Pu.1, acts in concert with the Gata1 transcription factor to regulate myeloid versus erythroid cell fate. Spi1 and Gata1 negative regulate each other’s activity in human myelo-erythroid progenitor cells as well as in zebrafish embryos, resulting in a myeloid cell fate when the balance is in favour of Spi1, while differentiation is directed towards an erythroid fate when Gata1 is the predominant factor [51]. Potential target genes of Spi1 regulation in zebrafish embryos were recently identified, resulting in novel marker genes for zebrafish myeloid cells [52]. At least two populations of myeloid cells can be distinguished in embryos by marker gene expression [52, 53]. The first population expresses the *csf1r* or *fms* gene coding for the macrophage colony stimulating factor (M-CSF) receptor. This gene retains macrophage-specific expression throughout development, but is also expressed in xanthophores [54]. The myeloperoxidase (*mpx*) gene is a specific marker of differentiated neutrophilic granulocytes, which are present from 2 dpf. These differentiated neutrophils are detectable by histochemical staining for Mpx enzyme activity and by the presence of Sudan-Black staining cytoplasmic granules [55, 56]. However, a distinct *mpx*-expressing myeloid population is also present at 1 dpf, prior to the appearance of differentiated neutrophils [52]. Expression of *mpx* in zebrafish embryos largely overlaps with expression of the lysozyme c (*lyz*) gene [53, 57, 58], while *csf1r* expression at 1 dpf overlaps with markers such as *cxcr3*.2, *mfap4*, *mpeg1*, and *ptpn6 *[52]. Partial overlap between some of these markers at later stages indicates the presence of multiple myeloid cell subsets that remain to be further characterized [52]. The putative zebrafish mast cells appear to form a distinct myeloid subset, which is characterized by carboxypeptidase 5 (*cpa5*) expression [59]. Between 2 and 3 dpf macrophages that have colonized the brain and retina undergo a phenotypic transition into early microglial cells and start expressing high levels of apolipoprotein-E, while simultaneously the expression of the common leukocyte marker L-plastin (*lcp1*) is down-regulated [60]. Expression of lymphoid markers, such as *lck* and *rag1*, in the developing thymus is detectable by 4 dpf [11, 34].

The primitive macrophages that arise from the anterior lateral plate mesoderm are capable of phagocytosing cellular debris, apoptotic cell corpses, and microbes, which can be injected into the blood circulation or into one of the closed body cavities, such as the hindbrain ventricle [15, 16]. Non-pathogenic infections, for example with *Escherichia coli* bacteria or with an LPS-mutant of *Salmonella enterica *serovar Typhimurium (*Salmonella typhimurium*), can be efficiently cleared by the embryonic immune system [15, 61]. Studies with different* Pseudomonas aeruginosa* mutant strains showed that the competency of the immune system to combat infections is increased at 2 dpf coincident with neutrophil differentiation [62]. Both macrophages and neutrophils were shown to migrate rapidly to sites of wound-induced inflammation or to sites of infection [15, 52, 55, 56, 58, 63-66]. These events can interfere with the normal routes of migration of the embryonic immune cells during development. For example, the colonization of brain tissues by macrophages that transform into microglia was shown to be disrupted during *Mycobacterium* *marinum* infection [63]. Furthermore, differentiated microglia appeared to leave the brain and join infection foci in the tissues. These studies and others discussed below have demonstrated the usefulness of the zebrafish embryo to study functions of innate immune cells in the context of a developing organism. In addition, hypersusceptibility of *rag1* mutant zebrafish to *M. marinum* infection has demonstrated that the role of the adaptive immune system can be studied in adult zebrafish [67]. 

## METHODS TO STUDY HOST-PATHOGEN INTERACTIONS IN ZEBRAFISH

### Routes of Infection

Adult zebrafish are usually infected by intraperitoneal or intramuscular injection, while embryos are systemically infected by microinjection directly into the blood circulation at 1-3 dpf, mostly using the posterior blood island or into the Duct of Cuvier, a wide blood circulation valley on the yolk sac connecting the heart to the trunk vasculature, as injection sites [3, 16, 68, 69]. Obviously, for high-throughput screening it would be more practical if infection could be accomplished simply by static immersion. Some examples of bacterial infection of adult zebrafish by bath immersion or by the combination of immersion with dermal abrasion have been described [70, 71]. Zebrafish larvae can be infected with bacterial pathogens via the oral route after opening of the mouth at 3 dpf, when also the colonization of the intestine by microbiota begins [5, 13, 72, 73]. Bacterial infection of 1-day-old zebrafish embryos using an immersion assay has thus far only been achieved with *Edwardsiella tarda*, a Gram-negative species that can cause a generalized septicaemia (edwarsiellosis) in many farmed fish species [70, 74], and with *Flavobacterium columnare*, another Gram-negative bacteria causing the highly contagious causes columnaris disease in cultured and wild fish populations worldwide [75]. However, in our experience mortality rates can be variable in immersion assays and expression of inflammatory marker genes can show large variation between individual embryos (unpublished results). Another route of infection suitable for high-throughput applications is injecttion into the yolk sac. Yolk infections with fast-replicating bacterial species resulted in massive bacterial growth and early lethality of the embryos [61, 69]. However, for slow-replicating species, such as *M. marinum*, yolk infection can be a useful system. We have found that yolk injection of *M. marinum* during the first hours of embryogenesis does not interfere with embryo development and that bacteria disseminate from the yolk into the tissues, where infected macrophages aggregate into early granulomas similar as upon the intravenous route of infection (unpublished results). An automated system for high throughput yolk injections is currently under development in our laboratory in collaboration with the company ZF-screens [8].

### Transgenesis

Transgenic reporter lines expressing fluorescent proteins under the control of leukocyte-specific promoters are valuable tools for studying host-pathogen interactions in the zebrafish model [18]. The Spi1 promoter has been used to generate transgenic lines that express green fluorescent protein (GFP) in early myeloid cells at 1 dpf [52, 76-78]. In addition, in fli1:EGFP transgenic fish that express GFP in the vascular system, early myeloid cells are also labeled [64]. The myeloperoxidase (*mpx*) promoter, which is specifically active in zebrafish neutrophils, has been used to generate two different transgenic lines. One of these lines was constructed by fusing a 8 kb promoter region to GFP [65], while the second line was constructed using a BAC recombineering strategy that better maintains the genomic structure [66]. Both lines faithfully label the neutrophil population with high GFP expression levels, but in the first line an additional population of cells with low GFP expression has been observed [58]. These cells were characterized as a population of inflammatory macrophages and can be distinguished from the neutrophil population not only by the GFP expression level but also by morphology, migratory characteristics and marker gene expression [58]. Recent live imaging studies of neutrophil motility illustrate the power of the zebrafish model in visualizing the dynamics of cell migration [79, 80]. Promoter fragments from the lysozyme C (*lyz*) gene have also been used to generate transgenic lines [81-83]. Although originally reported as a macrophage-specific marker, multiple reports have shown the overlap of *lyz* mRNA expression with that of the neutrophil marker *mpx *[53, 57, 58]. *Lyz:EGFP/DsRED2* lines display a large overlap of transgene expression with *mpx *expression in neutrophils, but labeling of a population of macrophages in these lines was also reported [81]. Other lines that label subsets of myeloid cells include the CGLY463 line, which has a YFP enhancer trap insertion close to a member of the *myc* gene family (*mych*) [57], and the MyD88:EGFP/DsReD2 lines, in which fluorescent protein expression is driven by the promoter of the MyD88 gene involved in innate immunity signaling [84]. Introduction of a membrane-bound GFP into the apolipo-protein-E (*apo-E*/*apoeb*) locus resulted in a transgenic line that labels zebrafish microglia [78]. Transgenic marker fish for T-lymphocytes, based on the *lck* promoter, are also available [85]. Other transgenic lines are eagerly awaited for, such as those expressing pan-leukocytic markers, like *lcp1* (*L-*plastin) or *ptprc* (CD45) [41], or macrophage-specific markers, like *csf1r *(*fms*) or the recently reported Spi1-dependent genes that are expressed in early macrophages [52]. In addition, transgenic reporter lines for activation of the immune response, such as a reporter line for NFκB activity, will be extremely useful [5].

The possibility to drive transgene expression in different leukocyte subsets is also useful for functional studies of genes involved in host-pathogen interactions and can be applied for expression of toxins to selectively ablate a specific cell type. The Gal4/UAS two-component system provides a highly versatile toolbox for transgene expression [86]. In this system a cell- or tissue-specific promoter is used to drive expression of the yeast Gal4 transcription factor. Such Gal4 driver lines can be crossed with lines expressing a transgene under control of the upstream activating sequence (UAS) of Gal4. A variety of UAS lines expressing different fluorescent proteins is available [86]. For example, a UAS:kaede line is very useful for lineage tracing studies by UV-mediated photoconversion of green-fluorescent kaede protein to the red fluorescent form. A UAS:nfsB-mCherry transgenic line can be used to drive expression of *E. coli* nitroreductase B, which can convert precursor drugs such as metronidazole (MET) into toxic cellular metabolites [86]. The cells that are targeted for ablation by nitroreductase B expression are simultaneously made visible due to the fusion of nitroreductase B with mCherry protein. The generation of Gal4 driver and UAS reporter lines has been boosted by the introduction of Tol2-based vectors that result in high rates of integration when co-injected with Tol transposase mRNA [87]. A potential drawback of the Gal4/UAS system may be that silencing of the UAS sequence might occur over subsequent generations, in which case frequent renewal of UAS lines would be necessary.

Transgene expression has also been used to generate zebrafish showing resistance to pathogenic infections. A zebrafish strain expressing the chicken lysozyme gene in epithelial tissues, liver and gill showed increased survival rates in infection experiments with lower doses of *F. columnare* and *E.* *tarda* [88]. Expression of antimicrobial peptides, including Tilapia hepcidin and epinecidin-1, also inhibited bacterial growth, specifically that of *Vibrio vulnificus* [89, 90]. Finally, transgenic zebrafish embryos expressing bovine lactoferricin were used as a food supplement enhancing resistance of wild type zebrafish adults to *E. tarda* infection [91]. 

### Gene Knockdown and Mutagenesis

The zebrafish model has advantages for both forward and reverse genetics approaches. Large scale forward genetic screens can be carried out with great efficiency and at relatively low costs since zebrafish can be kept at high population density and because of their fecundity. In forward genetics, a mutagenized fish population is screened for phenotypic alternations, for example increased susceptibility or resistance to an infection. The unbiased nature of such screens allows the identification of novel genes or novel functions for known genes. Germline mutations are most commonly introduced by ethylnitrosourea (ENU) treatment of male zebrafish, but retroviral- or transposon-mediated insertional mutagenesis strategies are also used [20]. ENU treatment results in random point mutations, which can be identified by positional cloning. The functions of numerous genes involved in vertebrate development have been identified through ENU mutagenesis screens in the zebrafish model [20]. The first successful application of this approach to the study of host-pathogen interactions was recently reported by the group of Lalita Ramakrishnan, who discovered a susceptibility locus for mycobacterial infection that is conserved between zebrafish and human (see below) [21].

Mutant populations generated for forward genetics screens are also useful for reverse genetics approaches. Genomic DNA from mutagenized fish can be screened by PCR amplification of genes of interest. The subsequent identification of mutations in PCR amplicons has become very efficient with the development of high-throughput sequencing methods. This approach, known as TILLING (Targeting Induced Local Lesions in Genomes), compensates for the fact that conventional knockout technology, as used in mice, is not yet available for the zebrafish [23]. The first zebrafish knockout mutant identified by TILLING was the mutant in the recombination activating *rag1* gene [92]. Homozygous *rag1* mutant fish are viable under normal culture conditions but showed hypersusceptibility to mycobacterial infection and are reported to die frequently upon fin clipping [9, 67, 93]. Enhanced transcription of complement and coagulation genes and increased abundance of neutrophils suggests that an enhanced innate immune response in *rag1* mutant fish may compensate for having a compromised adaptive immune system [93, 94]. Our preliminary analysis of a knockout mutant in MyD88, a key component of the innate immune response (see below), indicates a very low viability after the larval stage, suggesting that zebrafish rely heavily on an intact innate immune system for survival under standard culture conditions (unpublished results).

Although applications in the study of host-pathogen interaction have not yet been reported, the use of zinc-finger nuclease technology is a promising addition to TILLING approaches [95]. DNA-binding proteins containing three or more zinc-finger motifs are engineered to recognize unique target sequences in the genome. Due to fusion of the DNA-binding protein with the nuclease domain of Fok1, double stranded breaks can be generated at its target site. DNA break repair by the non-homologous end joining pathway will lead to stable gene disruption. The technology holds promises for adapting to knock-in approaches in zebrafish, whereby gene constructs are integrated into the genome based on homologous recombination.

Finally, a highly versatile method for reverse genetics in the zebrafish model is the use of antisense morpholinos [22, 24]. Morpholinos are stable synthetic oligonucleotides that can be designed to block translation or pre-mRNA splicing. Injection of morpholinos into zebrafish embryos at the 1-2 cell stage can result in a transient knockdown, which, dependent on the specific morpholino sequence and dose, can last up to the larval stage. In host-pathogen interaction studies, morpholino knockdown of the Spi1/Pu.1 transcription factor has been frequently applied [52, 62, 69, 96-98]. Due to the requirement of Spi1 for myeloid development, knockdown of this factor results in embryos that lack macrophages and show a major reduction of neutrophils [51, 53]. Conversely, morpholinos against the Gata1 transcription factor, an antagonist of Spi1 activity, can be used to expand the myeloid population [51]. Spi1 knockdown embryos (morphants) showed increased susceptibility to *P. aeruginosa* infection, while Gata1 morphants were less susceptible [62, 97]. The requirement of macrophages to contain bacterial growth was also demonstrated by infection of Spi1 morphants with *Staphylococcus aureus* [69]. In addition, the use of Spi1 morphants demonstrated that macrophages play an essential role in tissue dissemination of *M. marinum* infection [96]. Several applications of morpholino technology to study genes involved in the embryonic innate immune response are discussed below [21, 26, 52, 99-109].

### Assays and Reagents to Probe the Innate Immune Response

Reverse transcriptase PCR, microarrays and next generation sequencing studies have provided insights into the transcriptional response of zebrafish embryos, larvae, and adults to several types of infection (Table **1**, Fig. **2**). However, as the zebrafish is a relatively new model for infection studies, there is still a lack of many immunological reagents, such as antibodies for cell surface receptors and ELISA assays for cytokine activities. Fortunately, commercial investments in antibody production for zebrafish are increasing (e.g. www.anaspec.com). In some cases, antibodies for the mammalian orthologs of zebrafish proteins show cross-reactivity. For example, a polyclonal antibody against murine iNos (inducible nitric oxide synthase) proved useful to demonstrate colocalization of iNos protein with a subset of *M. marinum*-infected macrophages [103]. The product of iNos activity, nitric oxide (NO), can be visualized in living zebrafish embryos. In this bioassay, diaminofluorophore 4-amino-5-methylamino-2′-7′-difluorofluorescein diacetate (DAF-FM-DA) is used as a cell-permeant probe to detect sites of constitutive or inducible NO production [110]. A bioassay to measure production of reactive oxygen species (ROS) in whole zebrafish embryos or adult zebrafish kidney has also been developed [111]. This respiratory burst assay makes use of a non-fluorescent dye 2’,7’-dihydrodichloro-fluorescein diacetate (H_2_DCFDA) that is oxidized to dichlorofluorescein (DCF), a fluorescent product. Upon phorbol myristate acetate (PMA)-stimulation, zebrafish embryos at the age of 3 dpf produced ROS in enough abundance to be detected, but the response was more robust from 4 dpf [111]. A genetically encoded fluorescent sensor for hydrogen peroxide, HyPer, was recently used to show that a tissue-gradient of hydrogen peroxide functions to recruit leukocytes to wounds in zebrafish embryos [112]. This tool can now also be applied to visualize hydrogen peroxide production at infection sites. Finally, transient expression of an NFκB:luciferase reporter in zebrafish embryos has been applied to detect innate immune activation by microbial ligands [113].

Due to their transparency zebrafish embryos are highly suited for live imaging of phagocytosis and chemotaxis. The pHrodo *E. coli* Bioparticle conjugate (Molecular Probes, Invitrogen), which emits red fluorescence when inside the acidic environment of the phagosome, was used to demonstrate the phagocytic potential of MyD88-expressing leukocytes in zebrafish embryos [84]. In addition, LysoTracker and LysoSensor probes (Molecular Probes, Invitrogen) were elegantly used to image lysosomal acidification of microglia in the brain of zebrafish larvae [78]. The zebrafish embryo model is also useful for *in vivo* analysis of chemotaxis, following local injection of micro-organisms or compounds into the muscle tissue or into closed compartments, like the hindbrain ventricle, the otic vesicle or the pericardium [15, 16, 52, 56, 63, 96]. Due to their small size, the zebrafish model is less suitable for obtaining sufficient leukocytes for *in vitro* chemotaxis studies or other cell-based assays. However, it was recently shown that lavage of the coelomic cavity of adult zebrafish provides adequate cell numbers for immunological studies [114]. A good alternative is to take advantage of the common carp, a close relative of the zebrafish, for complementary *in vitro* studies. The carp has been widely used as an immunological model and the ontogeny of its innate immune system is highly similar to that of the zebrafish [115]. Based on transcriptome sequencing data, responses of carp and zebrafish embryos to mycobacterial bacterial infection were shown to be highly similar (our laboratory in collaboration with Ron Dirks, ZF-screens, unpublished results). The large size of adult carp permits to obtain abundant leukocyte populations from the blood by fluorescence activated cell sorting.

## PATTERN RECOGNITION RECEPTORS IN ZEBRAFISH

Recognition of pathogens is mediated by pattern recognition receptors (PRRs) of the innate immune system that are located on the cell surface, on endosomal compartments and in the cytosol [116]. The best studied family of PRRs is that of the Toll-like receptors (TLRs). The mammalian TLRs have specificity for a variety of conserved bacterial, fungal and viral ligands, while some members, such as TLR4, may also recognize endogenous danger-associated molecules produced during inflammation and infection [116]. Putative orthologs of the mammalian TLRs as well as fish-specific family members have been identified in zebrafish [117, 118] (Fig. **2**). Likely as the result of a genome duplication that has occurred during the evolution of teleost fish, zebrafish have two counterparts of some of the mammalian TLRs, for example there are two copies of the genes homologous to *TLR4* (*tlr4a*/*tlr4b*) and *TLR5* (*tlr5a*/ *tlr5b*). Whether this expansion at the level of the receptors is also associated with expanded ligand specificities has not yet been demonstrated. 

In contrast to the expansion of TLRs, there appears to have been a selection against the expansion of the down-stream components involved in TLR signal transduction. Zebrafish have single copies of the TLR adaptor molecules MyD88, Mal/Tirap, Trif/Ticam1 and Sarm, all expressed in embryonic leukocytes [84], while the fifth mammalian TLR adaptor Tram/Ticam2 appears absent from the fish lineage [118-120]. As another example, also Traf6, a central intermediate of TLR and TNF receptor signaling, occurs as a single copy in both mammals and zebrafish [120]. Synteny and phylogenetic analyses indicate that the zebrafish *tlr4a* and *tlr4b* genes are paralogous rather than orthologous to mammalian *TLR4 *[121]. Furthermore, lipopolysaccharide (LPS), the best studied ligand of mammalian TLR4, fails to stimulate the zebrafish Tlr4a/b receptors [105, 121]. This is consistent with the absence of the TLR4 co-receptors CD14 and MD2 in the zebrafish genome, which are required for LPS recognition. It is currently unknown whether recognition of other mammalian TLR4 ligands might be conserved between mammals and zebrafish. In the case of TLR5, the flagellin receptor, conserved ligand specificity between mammals and zebrafish does exist [26]. Furthermore, responsiveness of *tlr3* gene expression to viral infections, suggests a conserved role of this receptor in the recognition of viral RNA, which is further supported by the fact that poly(I:C) stimulation of HEK293 cells expressing zebrafish *tlr3* led to NFκB induction [74, 105, 122]. 

Triggering of the innate immune response in zebrafish embryos results in the transcriptional induction of well conserved transcription factors, such as members of the ATF, NFκB, AP-1 (JUN/FOS), IRF, STAT, ETS, MYC, MYB and C/EBP families [26, 30] (Fig. **2**), and the key signaling intermediates of the pathways leading to their induction in mammalian systems have also been identified in the zebrafish [120]. Knockdown analysis of Traf6 showed that a large set of genes depends on this central intermediate for induction or repression during *S. typhimurium* infection [123]. Knockdown of MyD88, which is the common adaptor of all TLRs except TLR3 in mammals, rendered zebrafish embryos more susceptible to infection with a normally non-pathogenic *S. typhimurium *LPS mutant strain [99]. Furthermore, as in mammals, the presence of MyD88-dependent and independent signaling routes, leading to interleukin 1 beta (*il1b*) and interferon (*ifnphi1*) induction respectively, was demonstrated [26]. The MyD88-dependent pathway was also shown to be required for the response of zebrafish larvae exposed to LPS and for the recruitment of neutrophils into the intestine in response to proinflammatory stimuli or in response to the endogenous microbiota that establishes the normal homeostatic level of intestinal neutrophils [100]. These results contrast with the TLR4- and MyD88-independent response that was reported for embryos microinjected with LPS [105]. In general, fish appear to be less sensitive to LPS than mammals [124], and it will require further study how signaling pathways mediating LPS responsiveness evolved in the fish lineage. G-protein-coupled receptor kinase 2 (Gprk2/GSK) was recently identified as a novel NFκB signaling regulator conserved between Drosophila and human, and its morpholino knockdown in zebrafish embryos blocked *E. coli*-induced *tnfa* and *il1b* expression [109]. However, it should be noted that fish have also evolved different routes to the induction of downstream target genes. In the Trif/Ticam1-dependent pathway to NFκB activation, the interaction of zebrafish Trif/Ticam1 with Tlr3, Tbk1 and Rip1 (Ripk1l) is conserved, but Trif/Ticam1 activates interferon in an IRF3/7-independent manner and does not interact with Traf6 like it does in mammals [119]. In addition, consistent with the absence of Tram/Ticam2, the Trif/Ticam1-dependent TLR4 pathway to interferon induction likely does not function in the zebrafish [125]. These observations help to distinguish general principles from species-specific mechanisms and to increase understanding of the evolution of the vertebrate immune system.

Other PRR families include the NOD-like receptors (NLRs), the RIG-I-like receptors (RLRs), the scavenger receptors, and lectins [116]. The canonical members of the mammalian NLR family, including Nod1, Nod2 and Nod3/ Nlrc3, are conserved in the zebrafish [120]. In addition, a subfamily of NLRs that resembles the mammalian NALPs, and a unique teleost NLR family have been identified [126]. There is evidence for the Spi1-dependent expression of at least one scavenger receptor (*LOC571584*, similar to macrophage receptor MARCO) in zebrafish embryonic myeloid cells [52], but many other family members of the scavenger receptors that are conserved in mammalian genomes can be identified in the zebrafish genome (unpublished). Also a soluble lectin encoding gene, *lgals9l1*, show enriched expression in embryonic myeloid cells and is dependent on Spi1 [52]. Polymorphisms in the zebrafish mannose-binding lectin (MBL) genes were associated with resistance to *Listonella anguillarum* [127]. One C-type lectin was recently proposed as the zebrafish ortholog of the dendritic cell-specific lectin DC-SIGN and showed inducible expression upon *Aeromonas hydrophila *infection [128]. Many similar C-type lectin encoding genes can be identified in the zebrafish genome some of which are highly inducible in infectious disease [25, 27] (unpublished results). Members of a family of immune-related, lectin-like receptors (illrs), showing structural similarity to mammalian C-type lectin natural killer cell receptors, are differentially expressed in the myeloid and lymphoid lineages [129]. In a family of 7 intelectins (X-lectins), one member (*zINTL-3*) was upregulated in adult fish tissues upon *Aeromonas salmonicida* infection [130].

From an evolutionary perspective, the peptidoglycan recognition proteins (PGRPs) are of special interest. In insects, these proteins trigger signal transduction pathways leading to production of antimicrobial peptides or digest biologically active peptidoglycan through their amidase activity. While amidase activity of mammalian PGRPs has also been demonstrated, several mammalian PGRPs lack this activity and have direct bactericidal functions [102]. Zebrafish PGRPs are unique compared to other vertebrate PGRPs in that they display both amidase and broad-spectrum bactericidal activities [102]. Morpholino knockdown analysis demonstrated an essential role for *pglyrp5* (*pgrp*-sc) in defence against *Salmonella enterica *and *Bacillus subtilis* infections of zebrafish embryos [102]. By RNAi suppression, a technique whose specificity in the zebrafish model remains controversial, the intracellular signalling pathways downstream of this gene were investigated [131]. The RNAi approach was also used to investigate *pglyrp6* (*pgrp6*) function, which was associated with susceptibility to *F. columnare* [75].

## OTHER RECEPTORS INVOLVED IN MICROBIAL CHALLENGE

Comprehensive analyses of cytokine and chemokine receptor families in zebrafish have been reported [120, 132], but few of the members have been functionally studied. The colonization of embryonic tissues by macrophages was shown to be dependent on the macrophage colony-stimulating receptor (Fms/Csf1r), but this receptor is not involved in the recruitment of macrophages to sites of infection [60, 63]. The zebrafish ortholog of the granulocyte colony-stimulating factor receptor (Gcsfr) and its ligand, granulocyte colony-stimulating factor (Gcsf, a class I cytokine), form a conserved signaling system involved in the production of myeloid cell lineages, both under homeostatic conditions and during emergency responses [104]. In Gcsfr morphants, primitive and definitive myelopoiesis were affected, resulting in reduced numbers of monocyte/macrophages as well as granulocytic cells. Emergency hematopoiesis triggered by LPS injection was defective in GCSFR morphants. Furthermore, the Gcsf/Gcsfr pathway was shown to be required for the migration of anterior myeloid cells during embryonic development, but not for the migration to wounding sites or for phagocytosis of bacteria.

The tumor necrosis factor receptor 1 gene (*tnfr1*/*tnfrsf1a*) was shown to be required for establishment of intestinal immune cell homeostasis upon microbiota colonization and for promoting intestinal inflammation in response to LPS treatment [100]. As mentioned above, these processes were also found to be MyD88-dependent, suggesting that TLR signaling functions upstream of TNF receptor signaling to regulate intestinal neutrophil influx [100]. TNF receptor signaling was also shown to mediate mycobacterial resistance in the zebrafish embryo model [103]. In TNF receptor morphants intracellular *M. marinum* growth and granuloma formation were accelerated, followed by necrotic death of macrophages and granuloma breakdown. While TNF has long been known as a key determinant in controlling tuberculosis [133], these results in the zebrafish embryo model provided direct evidence that TNF signaling is protective during the early stages of mycobacterial infection in the absence of adaptive immunity.

Many candidate receptors for class II cytokines, including the interferons, exist in the zebrafish genome [120]. The virus-induced fish interferons of the IFNφ family have been shown to constitute two subgroups that bind to two different receptor complexes. These complexes share a common short chain receptor (Crfb5) but differ in containing a distinct long chain receptor (Crfb1 or Crfb2) [101, 107]. While mammals have single interferon gamma receptors 1 and 2 genes (IFNGR1 and 2), zebrafish were reported to have two distinct IFNGR1 paralogs (*IFNGR1-1/crfb13* and *IFNGR1-2/crfb17*) that preferentially bind IFN-γ1 and IFN-γ2, respectively [134].

Among the chemokine receptors, only members of the CXC chemokine receptor family (CXCR) have been functionally studied in zebrafish. The SDF1-CXCR4 pathway plays an important role in the mammalian immune system and is required for several developmental processes in zebrafish embryos, including the migration of germ cells, neuronal cells and sensory cells of the lateral line organ [135]. Recently, CXCR4 was also implicated in neutrophil motility in zebrafish, in a study showing that constitutive SDF1-CXCR4 signaling induces the retention of neutrophils in hematopoietic tissue and impairs their trafficking to inflammation sites [80]. Zebrafish larvae exposed to AMD3100, a CXCR4 inhibitor, showed increased sensitivity to LPS toxicity [136]. Furthermore, in contrast to wild type larvae, larvae treated with AMD3100 or having a mutation in *cxcr4b* (*Odysseus* mutants) did not display LPS tolerance in response to pretreatment with sublethal doses [136]. In one-day-old zebrafish embryos only one *cxcr* receptor gene, *cxcr3.2*, was found to be specific for early myeloid cells and downstream of the Spi1 transcription factor [52]. Its expression overlapped largely with that of the macrophage specific marker *csf1r *and not with *mpx* expression. The *cxcr3.2* gene is most homologous to human *CXCR3* and *CXCR5*, which are expressed predominantly on T and B cells. Morpholino knockdown in zebrafish embryos showed that *cxcr3.2* is required for the migration of macrophages to a local site of *S. typhimurium* infection [52]. The zebrafish orthologs of the *CXCR1* and *CXCR2* receptor genes showed strong expression in the larval intestine [137].

An interesting class of fish-specific novel immune type receptors (NITRs) exists, which based on their sequence and structure have been proposed to represent the functional equivalents of mammalian natural killer receptors (NKRs) and to function within the innate immune system to regulate NK-cell-mediated cytotoxicity [138]. Consistent with this hypothesis, the NITRs have been shown to be expressed in the lymphocyte lineage, but not in the myeloid lineage [139].

Finally, like in mammals, four TCR loci (α, β, δ, and γ) are found in the zebrafish genome and the genomic organization of the TCRβ locus was recently described [140]. Analysis of TCRβ transcripts that had undergone VDJ recombination demonstrated that general locus organization and mechanisms used to generate junctional diversity are conserved between zebrafish and mammals.

## EFFECTORS OF THE INNATE IMMUNE RESPONSE

The expression of both proinflammatory (e.g. *il1b*, *tnfa*) and anti-inflammatory (e.g. *il10*) cytokine genes is induced when zebrafish embryos are systemically infected with a bacterial strain such as *S. typhimurium*, which rapidly proliferates and causes lethal infection [26] (Fig. **2**). Also systemic *P. aeruginosa* infection and static immersion in *E. tarda* suspension increased *il1b* and *tnfa* levels [62, 70]. In contrast to the situation in mammals, fish TNFα appears to have little effect on professional phagocytes. Instead, it has been shown to exert its main proinflammatory effects through the activation of endothelial cells and is thought to be predominantly involved in the recruitment of leukocytes rather than in their activation [141, 142]. 

The zebrafish chemokine family has undergone extensive expansion and diversification [143]. In some cases, such as for SDF1/CXCL12 and CXCL14, orthologies with the human system are clear, but for most members of the family orthologies are difficult to assign [143]. Interestingly, however, synteny analysis showed that zebrafish contains an ortholog (*il8*/*cxcl8*/*cxcl-C1a*) of human *IL8/CXCL8*, which is not present in the mouse [137]. Zebrafish* il8* is expressed in leukocytes and intestinal endothelial cells [137]. Its expression is up-regulated under inflammatory conditions caused by different bacterial infections [26, 137]. Expression of several other chemokines is also infection inducible, for example that of *cxcl-C1c* and *ccl-C5a* [26].

The virus-induced interferons (IFNs) in fish show a combination of features observed in mammalian type I (α and β) and type III (λ) IFNs and therefore have been named IFNφ [120]. Four zebrafish IFNφ genes (*ifnphi1/2/3/,4*) all induce the expression of antiviral genes, such as *viperin* and *mxa*, and two of them (*ifnphi1/2*) provided protection in a viral challenge assay [107]. The *ifnphi1* gene is also induced by *S. typhimurium* infection [26]. Injection of recombinant IFNφ1, IFNφ2, and IFNφ3 protected adult zebrafish from viral (SVCV) infection, and IFNφ1 protein also protected against *Streptococcus iniae* infection [144]. The type II (γ) IFNs of zebrafish are named IFN- γ1 (*ifng1-1*) and IFN-γ2 (*ifng1-2*). Recombinant IFN-γ2 was unable to protect adult zebrafish from viral and bacterial infections [144]. However, morpholino knockdown studies indicated partially redundant functions for the *ifng1-1 *and* ifng1-2 *genes in mediating resistance against *E. coli* and *Yersinia ruckeri* infections in zebrafish embryos [106]. In contrast, raising IFN-γ levels sensitized zebrafish embryos against bacterial infection, indicating the necessity of a tight control of IFN-γ levels [106].

We have observed that matrix metalloproteinase genes, specifically *mmp9* and *mmp13*, are among the strongest infection responsive genes in *S. typhimurium* infection as well as in several other types of bacterial infections in zebrafish embryos (Fig. **2**) [26] (unpublished results). MMPs can facilitate cell migration by degrading extracellular matrices but may also affect the activity of inflammatory molecules [145]. In situ hybridization studies detected *mmp9* and *mmp13* expression in embryonic myeloid cells [146]. Based on FACS sorting of different myeloid populations from an MPX:GFP transgenic line, *mmp9* and *mmp13* expression occurs in both the macrophage and neutrophil lineage [58]. Strong induction of these genes in epithelial cells was also observed around trauma sites following tail transaction [82, 146, 147]. Myeloid cell migration to such trauma sites was impaired by *mmp13* morpholino knock-down [82]. Expression of *mmp9* in response to flagellin and *S. typhimurium* treatment was shown to be downstream of the TLR5-MyD88 pathway, similar to the expression of *il1b *[26]. As discussed below, *mmp9* was recently shown to be required for recruitment of macrophages during mycobacterial granuloma formation [108]. Several other proteases, such as cathepsins and proteasome subunits, are also strongly induced during bacterial infections of embryonic and adult zebrafish [25-28, 30]. In addition, the expression of genes coding for acute phase proteins, such as complement components, fibrinogen, haptoglobin, hepcidin antimicrobial peptide, and serum amyloid A, is also markedly induced in both adult and embryo infection models (Fig. **2**) [25-30, 148, 149]. Finally, the bactericidal function of ROS production during infection is supported by morpholino knockdown of the NADPH oxidase family member dual oxidase (*duox*), which led to an impaired capacity of zebrafish larvae to control enteric *S. typhimurium* infection [150]. Knockdown of the cystic fibrosis transmembrane conductance regulator (*cftr*) gene also dampened ROS production in zebrafish embryos and led to an increased bacterial burden during *P. aeruginosa* infection [151].

## INSIGHTS INTO HOST-PATHOGEN INTERACTIONS FROM ZEBRAFISH BACTERIAL INFECTION MODELS

Over the recent years the number of zebrafish infection models for bacterial pathogens has rapidly increased (Table **1**). The role of bacterial virulence factors has been the focus of many investigations in both embryo and adult zebrafish infection models (Table **1**). Mutations in critical virulence determinants, for example type III secretion or quorum sensing, resulted in attenuated infections [62, 97, 152]. Large scale signature-tagged mutagenesis screens of *Streptococcus iniae* and *Streptococcus pyogenes* in adult zebrafish hosts proved a powerful strategy for the isolation of novel virulence determinants [153, 154]. A good example of exploiting transgenic zebrafish models is the use of fli1:EGFP zebrafish larvae to demonstrate that an extracellular sphingomyelinase of *P. aeruginosa* inhibits angiogenesis by selective cytotoxicity to endothelial cells [155]. Several groups have taken optimal advantage of the transparency of zebrafish embryos to study the interaction between intracellular bacterial pathogens and host phagocytes in real time [61, 63, 152, 156-158]. These studies have demonstrated that hallmarks of different host-pathogen interactions are reproduced in the zebrafish embryo model, such as the induction of granuloma formation by *M. marinum* and the formation of actin-based comet tails by *Listeria monocytogenes* bacteria following their escape from the phagosome into the cytosol [63, 158]. Below we highlight studies in the zebrafish-mycobacterium model that have led to important novel insights into the mechanisms of interaction between bacterial virulence factors and host determinants of the innate immune response.

### The Zebrafish -*Mycobacterium marinum* Model

*M. marinum* and *M. tuberculosis* are genetically closely related species that cause similar pathological hallmarks in their natural hosts, fish and human [159]. Both these pathogens survive within macrophages and induce the formation of granulomas, which are complex structures of infected and non-infected immune cells that provide a niche for the long term persistence of mycobacteria inside their host [1, 159]. The structure of *M. marinum*-induced granulomas in adult zebrafish strongly resembles that of human tuberculous granulomas, including the presence of a necrotic (caseous) center, which is not observed in the mouse model of tuberculosis [67, 160]. Superinfecting *M. marinum* bacteria were shown to traffic rapidly into pre-existing granulomas and reach their caseous lesions [161]. As in human tuberculosis, maximal control of *M. marinum* infection in zebrafish is dependent on an intact adaptive immune system [67]. Based on genetic differences and virulence *M. marinum* strains can be divided into two distinct types, one causing an acute disease in adult zebrafish with early lethality, and the second causing a chronic disease with granuloma formation in different organs and survival of the animals for at least 4 to 8 weeks [162]. While the two types of strains evoked partially overlapping host transcriptome signatures, strong differences in the host response were also observed [27]. Within one day after infection, zebrafish infected with an acute-disease causing strain showed higher expression of genes encoding MHC class I proteins, matrix metalloproteinases, transcription factors, cytokines and other common immune response proteins. In contrast, small GTPase and histone gene groups showed higher expression in response to infection with a strain causing chronic disease. Deep sequencing analysis further demonstrated the complexity of the host response to *M. marinum*, including the infection-dependent induction of different transcript isoforms [28]. A substantial overlap between the transcriptome signatures of *M. marinum*-infected zebrafish adults and embryos indicates a major contribution of the innate component of the immune system in the response to mycobacterial infection [27].

**Fig. (3):**
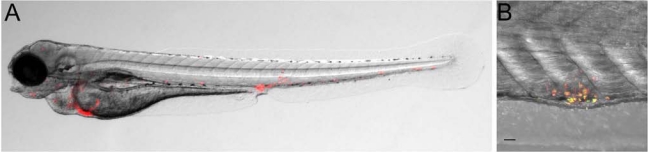
*M. marinum* infection of zebrafish embryos. (**A**) Fluorescence and bright-field overlay image of a zebrafish larva at 5 dpf showing the formation of granuloma-like cell aggregates containing red fluorescently labeled *M. marinum* bacteria. (**B**) Confocal microscopy and bright-field (DIC) overlay image of a granuloma-like aggregate in the tail of a zebrafish larva at 7 dpf. *M. marinum* bacteria in the aggregate show expression of a constitutive mCherry reporter and a granuloma-activated GFP reporter (GFP driven by the gag7 (*granuloma-activated gene 7*) promoter [63]. Embryos were infected by injection of *M. marinum* bacteria into the blood island at 1 dpf. Scale bar 25 µm.

Pioneering work of the group of Lalita Ramakrishnan demonstrated that the initial stages of granuloma formation can be studied in the zebrafish embryo model [63, 163]. Following intravenous injection of *M. marinum* bacteria at 1 dpf, tight aggregates of infected and non-infected macrophages are observed within several days (Fig. **3**). Macrophages in these aggregates adopt a distinctive morphology [63]. Furthermore, granuloma-activated genes (*gag* genes) of *M. marinum*, which are genes that are only activated when the bacteria are contained inside a granuloma, are also activated in these embryonic macrophage aggregates (Fig. **3**) [63]. These results led to the important conclusion that the context of the innate immune system is sufficient to initiate granuloma formation [63]. Thus, while T-lymphocytes form an important component of the mature granuloma, the initial formation of granulomas is independent of these cells. Subsequent work of the Ramakrishnan laboratory shed light on the role of the macrophage in early mycobacterial infection [96]. It has been debated whether macrophages control mycobacterium infected only after activation by the adaptive system, or that macrophages control infection from early on. Infection studies in embryos that lack macrophages due to knockdown of the Spi1/Pu.1 transcription factor showed that macrophages do restrict the growth of *M. marinum*, but also are essential for spreading of *M. marinum* into embryonic tissues. Therefore, although macrophages might not be regarded as an optimal growth niche, the residence of mycobacteria in these cells can be viewed as an evolutionary specialization that provides a mechanism for rapid intracellular spreading and tissue dissemination [96].

By studying the function of the mycobacterial RD1/ESX-1 virulence locus important novel insights were obtained into the role of granuloma formation during the early stages of infection [108, 157, 164]. The RD-1/ESX-1 locus is conserved between *M. tuberculosis* and *M. marinum* and essential for complete virulence of both species [159]. The locus is deleted in *M. bovis* BCG, which is the human vaccine strain but is only partially protective. It encodes a secretory apparatus and secreted proteins, including ESAT-6 and CFP-10. RD1 deletion impairs cell to cell spreading of *M. tuberculosis* in cultured cells [165]. In zebrafish embryos, infection with *M. marinum* RD1 mutant bacteria (ΔRD1) is attenuated compared to infection with wild type bacteria. Simultaneously, the formation of granulomas is reduced in ΔRD1 infection, while growth inside macrophages is unaffected [164]. In contrast, *M. marinum* mutated in the *erp* locus, which is required for cell wall integrity, have a growth defect inside macrophages [166]. Detailed *in vivo* imaging studies of the infection of zebrafish embryos with wild type and mutant bacteria subsequently demonstrated that the RD1 locus is required for the recruitment of new macrophages to granulomas and for the motility of macrophages inside granulomas [156]. This motility of newly arriving macrophages was shown to be required for efficient phagocytosis of infected macrophages undergoing apoptosis. In embryos infected with wild type mycobacteria, this mechanism leads to a rapid expansion of infected macrophages, some of which escape from the primary granuloma to seed secondary granulomas [156]. The mechanistic basis of RD1-dependent granuloma formation was subsequently clarified [108]. The secreted protein ESAT-6 was found to induce *mmp9* expression in epithelial cells neighboring infected macrophages. Morpholino knockdown analysis next showed that disruption of *mmp9* function impaired the recruitment of macrophages into granulomas and attenuated bacterial growth. Taken together, these studies in the zebrafish embryo model have provided strong evidence that mycobacteria actively exploit the early stages of granuloma formation for their local expansion and dissemination, which has challenged the traditional view of the granuloma as a host protection barrier.

A forward genetic ENU mutagenesis screen in zebrafish larvae yielded three classes of mutants with distinct patterns of innate susceptibility to *M. marinum* infection [21]. The first class showed reduced granuloma formation similar as observed in infections with ΔRD1 mutant bacteria, the second class displayed resistance to infection prior to granuloma formation, while the third class displayed hypersusceptibility phenotypes. For mapping a mutation of the hypersusceptibility class, Tobin *et al.* took advantage of the distinctive bacterial cording phenotype displayed by virulent mycobacteria in the extracellular environment [21]. The mutation was mapped in the region of the leukotriene A_4_ hydrolase (*lta4h*) gene, likely in a regulatory region. Analysis of an additional retroviral insertion mutant together with morpholino knockdown analysis confirmed that the *lta4h* locus modulates susceptibility to mycobacterial infection. The Lta4h enzyme catalyzes the final step in the biosynthesis leukotriene B_4 _(LTB_4_), an eicosanoid with potent proinflammatory and chemoattractive properties. When *lta4h* is mutated, the substrate of Lta4h activity, leukotriene A_4 _(LTA_4_), is redirected to the production of lipoxin A_4 _(LXA_4_), which has anti-inflammatory properties. The balance between the eicosanoid LTB_4_ and the lipoxin LXA_4_ determines TNF production, a critical component of resistance to mycobacterial infection in both mammals and zebrafish embryos [103, 133]. LTB_4_ injection induced *tnf *expression in wild type embryos, while *lta4h* mutants showed reduced *tnf *mRNA levels. Conversely, LXA_4_ injection decreased *tnf *expression, and the high levels of this anti-inflammatory lipoxin appear to be the predominant cause of the increased mycobacterial growth in *lta4h* mutants. In the model proposed by Tobin *et al*., an optimal balance between LTB_4_ and LXA_4_ production will lead to a level of TNF production that is sufficient to control infection but not high enough to induce excessive inflammation and tissue damage [21]. Interestingly, the authors also found that heterozygosity at the *LTA4H* locus is correlated with susceptibility of human populations to two mycobacterial diseases, tuberculosis and leprosy. This is an excellent example of the potential of the zebrafish model to discover host pathogen-interaction mechanisms relevant to human infectious diseases.

## PROSPECTS FOR HIGH-THROUGHPUT ANTIMICROBIAL DRUG SCREENS

Zebrafish infection models developed over the last decade have many advantages for studying the vertebrate host immune response and the interaction with bacterial virulence factors. In particular, the combination of *in vivo* imaging and genetic analysis in zebrafish embryo and larval models is extremely powerful. The use of zebrafish embryos and larvae for drug screens is also highly attractive [2]. Screens can conveniently be performed in 96-wells plates and embryos easily take up compounds through the skin. For example, addition of anti-pseudomonad antibiotics to the embryo medium could rescue zebrafish embryos from lethal *P. aeruginosa* infection [62]. The bottleneck for antimicrobial drug screens in zebrafish embryos or larvae has been the development of high-throughput infection systems. As discussed above, for the majority of bacterial pathogens tested, infection of embryos by static immersion proved ineffective and microinjection was required. The development of an automated system for yolk injection of embryos with *M. marinum* is an important step towards high-throughput applications [202]. 

A major advantage of performing drug screens in the context of a developing vertebrate organism is that information on teratogenicity or general toxicity of compounds can be obtained simultaneously with drug efficacy testing. In the case of antimicrobial drug screens there are additional advantages to the use of an *in vivo* system. First of all, the physiological status of bacteria when inside their host is usually very different from that under cultured conditions, and infections of human or animal cultured cells cannot mimic the disease symptoms of an *in vivo* infection. As a result, drugs that inhibit bacterial growth *in vitro* may be ineffective *in vivo*. Conversely, drugs that are only effective *in vivo* cannot be identified by *in vitro* screens. Secondly, the use of an *in vivo* screening system makes it possible to focus the search for novel antibiotics also on targeting host-specific pathways. This is especially important in the case of intracellular pathogens, such as mycobacteria, that manipulate the host’s immune system in an intricate manner. Several recent studies underscore the potential for immuno-modulatory drugs or drugs that target host pathways manipulated by mycobacteria [21, 108, 167, 168]. For example, host factors involved in phagosome maturation or in the regulation of autophagy may be attractive targets for the development of drugs against tuberculosis [167, 168]. Supported by studies in the zebrafish model, interception of host MMP9 production or the use of drugs that modulate the effect of anti-inflammatory lipoxins might be promising strategies for developing tuberculosis therapies [21, 108]. 

In conclusion, high-throughput drug screens in zebrafish embryos and larvae hold much promise to fill the gap between cell culture-based screens and screens in rodent models. In addition, this transparent model may also prove useful for drug administration and drug trafficking studies. 

## Figures and Tables

**Table 1:** Study of Bacterial Virulence Genes and Host Immune Response Genes Using Adult, Embryonic and Larval Zebrafish Models

Bacterial species	Infection models	Study of virulence genes*	Expression of host immune response genes**	Functional studies of host genes***
*Aeromonas hydrophila*	adult [171] larvae [13]		qPCR [13, 171-173]	
*Aeromonas salmonicida*	adult [148]		qPCR [130, 148]	
*Aeromonas veronii*	larvae [174]			
*Bacillus subtilis*	embryo [15, 102]			antibacterial function of pglyrp5 [102]
*Bacillus sphaericus*	adult [175]			
*Bacillus thurigiensis*	adult [175]			
*Burkholderia cenocepacia*	adult [176] embryo [151, 152]	BDSF quorum sensing [176] AHL quorum sensing [152]		
*Edwardsiella ictaluri*	adult [177]			
*Edwardsiella tarda*	adult [70, 88, 91, 178] embryo [70, 151]	Type VI secretion system [178]	qPCR [70, 74]	transgenic expression of bovine lactoferrin [91] transgenic expression of chicken lysozyme
*Escherichia coli*	embryo [15, 61, 98, 109, 151] larvae [73, 172]	StcE (pO157) secreted protease [73] HlyA and CNF1 toxins and other factors of extraintestinal pathogenic (ExPEC) strains [98]	qPCR/RT-PCR [106, 109, 172]	overexpression and knockdown of IFN- γ1/2 [106] myeloid cell depletion by Spi1/Pu.1 knockdown [98] knockdown of Gprk2/GRK5 NFkB signaling regulator [109]
*Flavobacterium columnare*	adult [88, 179, 180] embryo [75]		qPCR [75, 179]	RNAi suppression of pglyrp6 [75] transgenic expression of chicken lysozyme [88]
*Flavobacterium johnsoniae*	adult [180]			
*Francisella sp.*	adult [181]		qPCR[181]	
*Haemophilus influenzae*	embryo [151]			
*Leptospira interrogans*	embryo [156]			
*Listeria monocytogenes*	adult [182] embryo [158]	hly listeriolysin [158] actA actin tail recruitment [158]		
*Listonella anguillarum*	adult [127, 149] larvae [137]		qPCR [137, 149]	resistance correlation of MBL haplotypes [127]
*Mycobacterium marinum*	adult [67, 160, 161] [71, 162, 183] embryo [63]	RD1/ESX1secrection system [67, 164, 166] [108, 157] extRD1 (extended RD1) [183] erp cell wall integrity locus [21, 96, 103, 166] iipA/iipB locus for invasion and intracellular persistence [184]	in situ [103, 108] qPCR/RT-PCR [108, 118] microarray [25, 27] deep sequencing [28]	role of macrophages by Spi/Pu1 knockdown [96] role of TNF signaling in resistance [103] mmp9 function in granuloma formation [108] function of lta4h susceptibility locus [21]
*Mycobacterium peregrinum*	adult [71]			
*Pseudomonas aeruginosa*	embryo [62, 97, 151, 155, 185] larvae [13]	Type III secretion system [62, 97, 186] Flagellar apparatus [186] Quorum sensing [62, 151] PlcHR sphingomyelinase [155] PUMA3 extracytoplasmic function sigma factor [185]	qPCR [13, 62, 172, 186]	manipulation of myeloid cell numbers by the Spi1/Gata1 balance [62, 97] specific *P. aeruginosa* resistance mediated by cystic fibrosis transmembrane conductance regulator (*cftr*) gene [151]
*Pseudomonas fluorescens*	larvae [174]			
*Salmonella arizonae*	embryo [63]			
*Salmonella enterica* serovar Typhimurium (*Salmonella typhimurium*)	embryo [61, 102] larvae [150]	Ra LPS O-antigen [26, 61, 99]	in situ [26, 150] qPCR [26] microarray [26, 123] deep sequencing [30, 123]	MyD88 signaling function [26, 99] Traf6 signaling function [123] TLR5 function in flagellin recognition [26] antibacterial function of pglyrp5 [102] cxcr3.2 function in macrophage migration [52] antibacterial function of duox [150]
*Staphylococcus aureus*	adult [148, 187] embryo [69, 151]	PerR, PheP, SaeR and other [69]	qPCR [148]	myeloid cell depletion by Spi1/Pu.1 knockdown [69]
*Streptococcus agalactiae*	adult [89, 90]			transgenic expression of antimicrobial peptides [89, 90]
*Streptococcus iniae*	adult [68, 141, 188, 189]	capsule formation and other virulence genes from signature-tagged transposon mutagenesis [153] capsule formation [190] M-like protein [191] C5a peptidase [191]	qPCR [144]	injection of recombinant interferon proteins [144] injection of recombinant TNF and TNF plasmid [141]
*Streptococcus pyogenes*	adult [68, 188, 189, 192] larvae [192]	silB/silC quorum sensing and other virulence genes from signature-tagged transposon mutagenesis [154] MtsR iron uptake regulator [193] Siu iron uptake transporter [194] biofilm formation [195] Shr surface protein [196] signal recognition particle pathway [197] streptolysis S cytolytic toxin [192] glutathion peroxidase [198] SalY ABC transporter (salivarcin A lantibiotic locus) [199]		
*Streptococcus suis*	adult [200]	novel infection-related factor Trag [201]	microarray [29]	
*Vibrio anguillarum*	larvae [72]			
*Vibrio vulnificus*	adult [89, 90]			transgenic expression of antimicrobial peptides [89, 90]
*Yersinia ruckeri*	embryo [106]		RT-PCR [106]	overexpression and knockdown of IFN gamma1/2 [106]

* Studies of bacterial virulence genes using zebrafish infection models; studies of zebrafish exposed to bacterial toxins are not included.

** Analysis of the zebrafish host transcriptome response to bacterial infections by mRNA in situ hybridization (in situ), reverse transcriptase PCR (RT-PCR), quantitative RT-PCR (qPCR), microarray or deep sequencing; studies using heat-killed bacteria or bacterial ligands are not included.

*** *In vivo* functional studies of zebrafish genes involved in the host response to bacterial infection; *in vitro* studies in zebrafish cell cultures are not included.
